# Neuron‐inducing therapy using embryonic neural progenitor cells embedding positively charged gold nanoparticles in rats with complete spinal cord injury

**DOI:** 10.1002/ctm2.981

**Published:** 2022-07-15

**Authors:** Gong H. Han, Wan‐Kyu Ko, Seong J. Kim, Daye Lee, Dabin Jeong, Inbo Han, Seung H. Sheen, Seil Sohn

**Affiliations:** ^1^ Department of Neurosurgery CHA Bundang Medical Center CHA University Seongnam‐si Gyeonggi‐do Republic of Korea; ^2^ Department of Life Science CHA University Seongnam‐si Gyeonggi‐do Republic of Korea; ^3^ Department of Biology Lawrence University Appleton Wisconsin USA

Dear Editor,

Traumatic spinal cord injury (SCI) results in neuron loss and axon degeneration at the injury area.^1^ Embryonic‐spinal‐cord‐derived neural progenitor cells (NPCs) have been highlighted given that NPCs can differentiate into neurons.^2^ However, under SCI most NPCs undergo differentiation into astrocytes rather than into neurons.^3^ Glial fibrillary acidic protein (GFAP) barriers from activated astrocytes following SCI disrupt the recovery of damaged neurons/axons in injured areas.^4^ We aimed to improve injured neurons using NPCs embedding positively charged gold nanoparticles (GNPs) in complete SCI rats.

GNPs have the unique characteristics of being non‐toxic, non‐immunogenic and biocompatible, which make them attractive materials for biomedical applications.^5^ GNPs have also been successfully employed to induce differentiation into neurons in mouse embryonic stem cells in vitro.^6^ GNPs are classified as either negatively charged GNPs (nGNPs) or positively charged GNPs (pGNPs) according to their surface charge. To measure the size of GNPs, absorbance was investigated. Here, the maximum absorbance of nGNPs and pGNPs was found at a wavelength of 526 nm (Figure 1A). The diameters of both nGNPs and pGNPs were 32 nm (Figure 1B) and the corresponding surface charges were −33.05 ± .81 and 49.35 ± 1.14 mV (Figure 1C).

**FIGURE 1:**
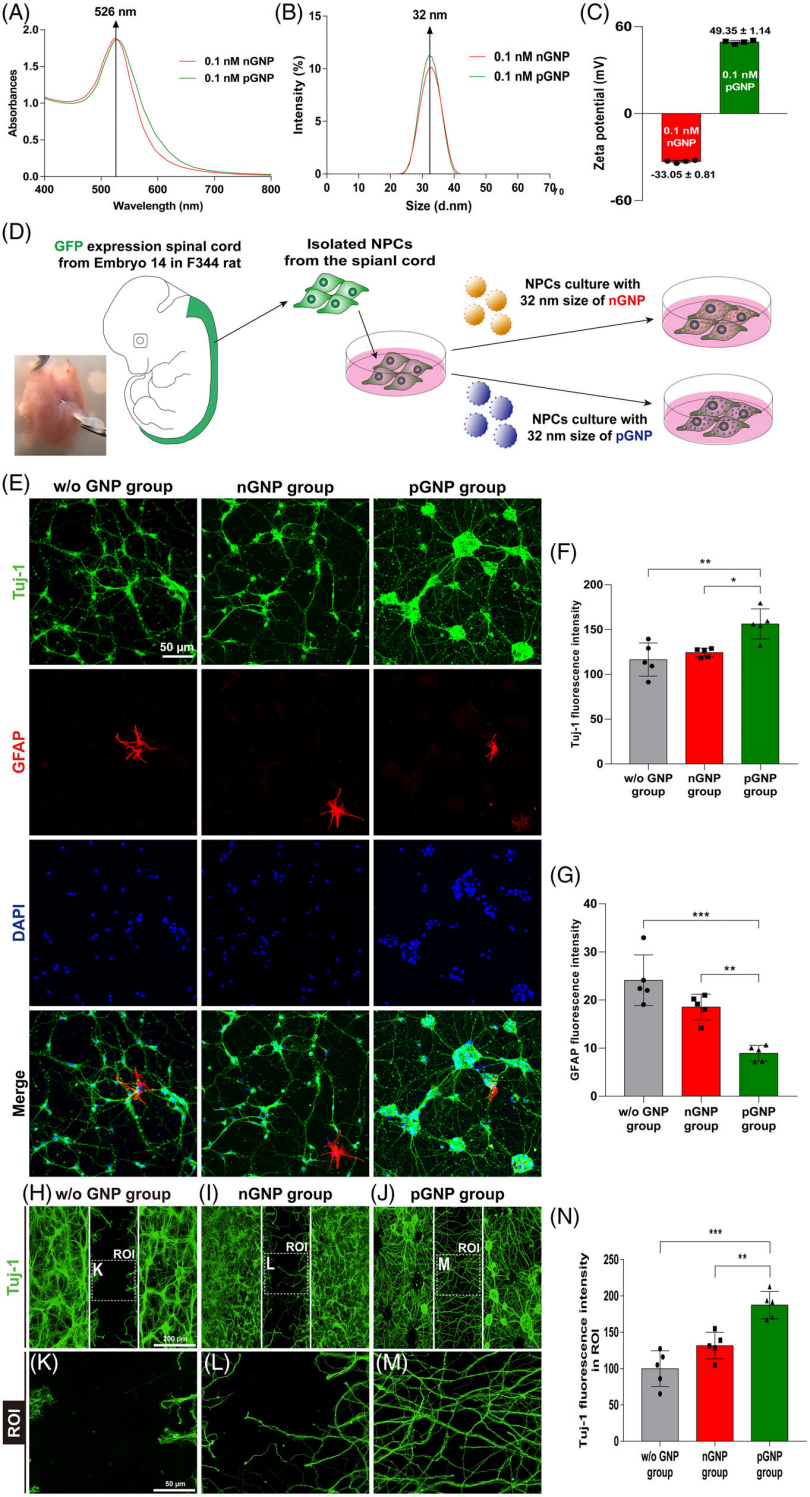
(A) Absorbance levels ranging from 400 to 800 nm of negatively charged gold nanoparticles (nGNPs) and positively charged gold nanoparticles (pGNPs). (B) Size distributions of the nGNPs and pGNPs analysed by dynamic light scattering (DLS). (C) Surface charges of the nGNPs and pGNPs. Cell culture processes of isolated embryonic neural progenitor cells (NPCs) expressing green fluorescent protein (GFP). (D) Schematic procedure for the preparation of the nGNP/pGNP groups. NPCs were cultured for 7 days without GNP (w/o GNP), with nGNPs, or with pGNPs. (E) On day 7, the NPCs were co‐stained with glial fibrillary acidic protein (GFAP; expressed in red), neuron‐specific beta‐III tubulin (also called Tuj‐1; expressed in green) and DAPI (expressed in blue). The fluorescence intensities of (F) Tuj‐1 and (G) GFAP were divided according to the DAPI intensities for quantification. The neuronal growth of NPCs towards the block region. (H–J) Neuronal growth in the w/o GNP group, nGNP group and pGNP group after 2 days of cell culturing. (K–M) High‐magnification images of the region of interest (ROI) from (H–J). (N) Quantitative analysis of the Tuj‐1 intensity from (K–M). Results are expressed as the mean ± SEM (*n* = 5/group, ^*^ *p* < .05, ^**^ *p* < .01 and ^***^ *p* < .001)

We found that both GNPs are non‐toxic to NPCs (Figure S1). We also found that both types (nGNPs/pGNPs) can be attached to the cell surface for the endocytosis (Figure S2). We found that most of the embryonic‐spinal‐cords‐derived cells were green fluorescent protein (GFP)‐positive (Figure S3A). GFP‐expressing cells were stained with Pax6 and Sox2 markers to show a ratio of NPCs to each marker (Figure S3B–D). Isolated NPCs were cultured with a .1‐nM concentration of nGNPs or pGNPs for 48 h (Figure 1D) to investigate the cellular differentiation. Class III beta‐tubulin (also called Tuj‐1, a neuronal/axonal marker)^7^/GFAP (an astrocyte marker) stained images are presented in Figure 1E. The Tuj‐1 intensity in the pGNPs was increased relative to the corresponding levels in the w/o GNP and nGNPs cases (Figure 1F). The GFAP intensity in the pGNPs was decreased compared to those in the w/o GNP and nGNPs cases (Figure 1G). Neurite growth was also assessed using NPCs and GNPs (Figure 1H–M). The Tuj‐1 intensity within block regions in the pGNPs was increased compared to those in the w/o GNP and nGNP cases. Taken together, among the groups of pGNP‐treated NPCs, nGNP‐treated NPCs and non‐GNP‐treated NPCs (w/o GNP), the pGNP‐treated NPCs showed greatest differentiation into neurons and lowest into astrocytes. Afterwards, we focused on the neuron‐inducing effect of pGNPs in an in vivo test.

The overall process of the in vivo evaluation is shown in Figure 2A. The pre‐determined time points, the complete compression SCI method and the three experimental groups used here (Injury only [Injury group], Injury + NPC [NPC group] and Injury + NPC + pGNP [NPC‐pGNP group]) are shown. A hydrogel that consists of glycol chitosan (gC) and oxidized hyaluronate (oHA) was used as an NPC carrier. This gC–oHA (CHA) gel has several advantages, the most important being that it is fully degradable in an injured spinal cord.^8^ We transplanted GFP‐expressing NPCs into three sites of the spinal cord for the NPC and NPC‐pGNP groups (Figure 2A). Multiple injections around the injury epicentre were employed in clinical trials.^9^ The white dashed lines indicate injured areas that are surrounded by astrocytes (Figure 2B–D). We were able to find surviving NPCs (stained with GFP) within the injured areas of the NPC (Figure 2C) and NPC‐pGNP groups (Figure 2D). We stained the NPC‐transplanted regions with neuronal nuclei (NeuN)/GFP markers to quantify cells differentiated into neuronal cells (stained with NeuN) from NPCs (stained with GFP). The NeuN/GFP outcomes in the NPC and NPC‐pGNP groups are shown in Figure 2E–H. The NeuN/GFP area (expressed as yellow) in the region of interest (ROI) is indicated with arrows in merged images (Figure 2G,H). The NeuN/GFP intensity in the NPC‐pGNP group exceeded that of the NPC group (Figure 2I).

**FIGURE 2:**
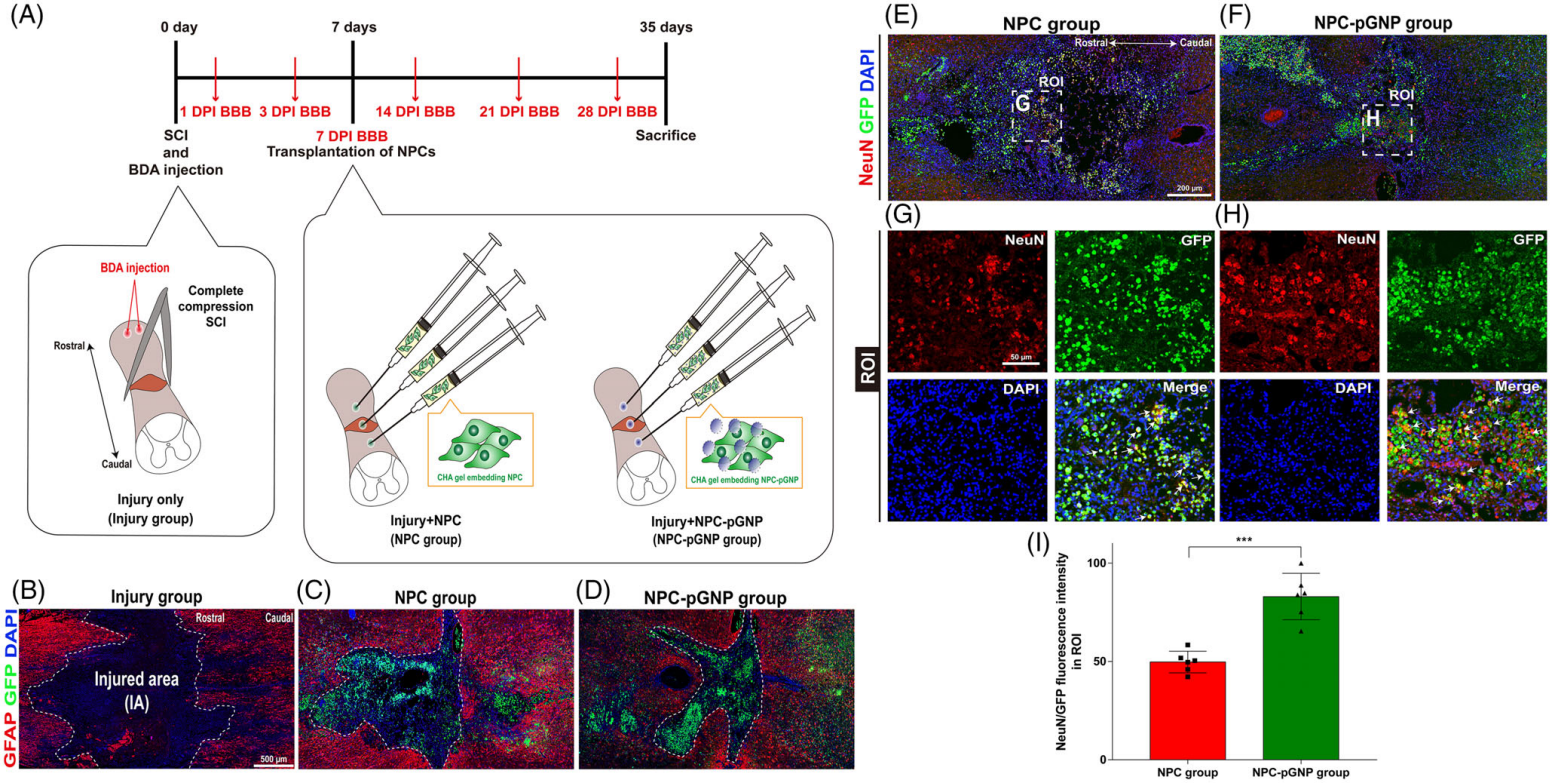
Schematic procedures from spinal cord injury (SCI) to sacrifice and confirmation of living neural progenitor cells (NPCs) in vivo: (A) schematic procedures for the injury group (injury only), NPC group (injury + glycol chitosan and oxidized hyaluronate [CHA] gel embedding NPCs) and NPC‐positively charged gold nanoparticles (pGNP) group (injury + CHA gel embedding NPCs/pGNPs. (B–D) Immunofluorescence (IF) analysis through glial fibrillary acidic protein (GFAP)/green fluorescent protein (GFP)/DAPI staining in injured regions of SCI rats. Biotinylated dextran amine (BDA) was injected into SCI rats on day 0. Neuronal differentiation of transplanted NPCs in the NPC and NPC‐pGNP groups. (E and F) Representative images showing neuronal nuclei (NeuN)/GFP/DAPI in the NPC and NPC‐pGNP groups. (G and H) High‐magnification images of the region of interest (ROI) from (A and B). (I) Quantitative analysis of the NeuN intensities from (G and H). NeuN intensities are divided according to GFP intensities for quantification. Results are expressed as the mean ± SEM (*n* = 6/group, ^***^ *p* < .001)

GFAP‐stained barriers around lesions are indicated by white dashed lines (Figure 3A–F). The rostral areas around the lesions are stained with GFAP/Tuj‐1/DAPI markers. The Tuj‐1 intensity in the NPC‐pGNP group was increased compared to those in the Injury and NPC groups (Figure 3G). The NPC‐pGNP group had the lowest GFAP intensity (Figure 3H). We stained the NPC‐transplanted regions with neuronal differentiation makers, including Pax6, Nestin and NeuroD1 (Figure S4A–C). The Pax6, Nestin and NeuroD1 intensities in the NPC‐pGNP group were increased compared to those in the Injury and NPC groups (Figure S4D–F). The Tuj‐1 and NF‐H mRNA of the NPC‐pGNP were increased compared to those of the Injury and NPC (Figure S5A,C and Table S1). GFAP mRNA of the NPC‐pGNP was decreased compared to that of the Injury and NPC (Figure S5B). Biotinylated dextran amine (BDA) and Tuj‐1 are widely used for axon detection in spinal cords.^2^, ^10^ The caudal areas around the lesions were stained with BDA/GFAP/DAPI markers (Figure 3I–N). The BDA intensity in the NPC‐pGNP group was increased relative to those in the Injury and NPC groups (Figure 3O). We investigated whether the transplantation of NPCs embedding pGNPs could improve the motor function in SCI rats (Figure 3P). One day after SCI, the locomotor scores of the injured rats were 0 or 1 in all groups. On day 35, the score of NPC‐pGNP group showed significant improvement compared to the score of NPC group.

**FIGURE 3:**
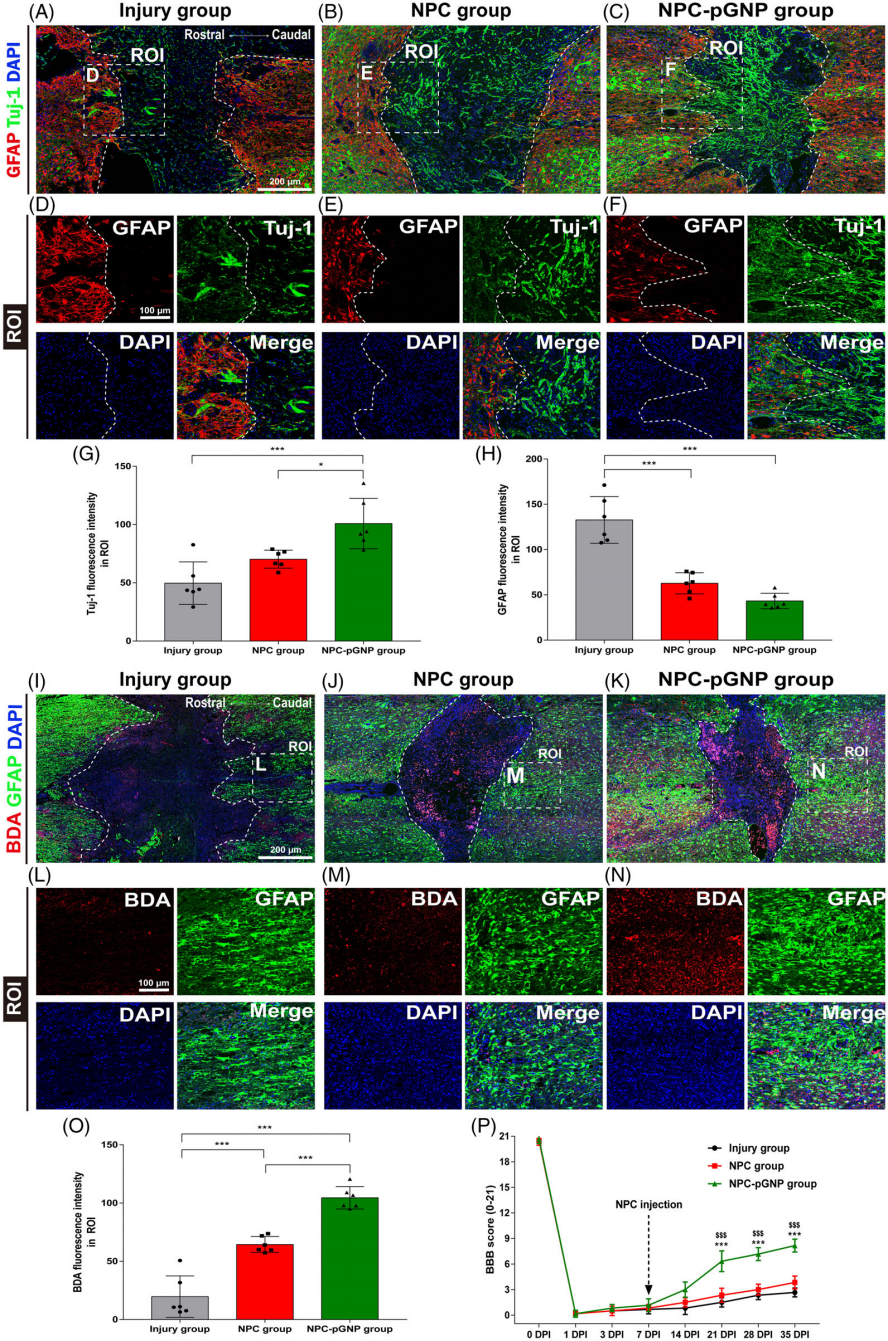
Immunofluorescence (IF) staining and measurements of Basso, Bettie and Bresnahan (BBB) locomotor scores for the evaluation of recovery in spinal cord injury (SCI) rats. Representative images in the (A and I) injury group, (B and J) neural progenitor cell (NPC) group and (C and K) NPC‐positively charged gold nanoparticles (pGNP) group of SCI rats. (D–F) High‐magnification images of the region of interest (ROI) from (A–C). (L–N) High‐magnification images of ROI images beyond the epicentre lesion from (I–K). (G) Quantitative analysis of the Tuj‐1 intensity in the ROI. (H) Quantitative analysis of the glial fibrillary acidic protein (GFAP) intensity in the ROI. (O) Quantitative analysis of the biotinylated dextran amine (BDA) intensity in the ROI. Results are expressed as the mean ± SEM (*n* = 6/group, ^*^ *p* < .05 and ^***^ *p* < .001). (P) Locomotor scores were evaluated in the injury, NPC and NPC‐pGNP groups for 35 days after SCI. Differences between the groups of NPC and NPC‐pGNP, shown as mean ± SD: ^***^ *p* < .001. Differences between the group of Injury and NPC‐pGNP, shown as mean ± SD: ^$$$^ *p* < .001

In summary, pGNPs increased neuronal differentiation (Figure 1F)/growth (Figure 1N) of embryonic NPCs in vitro. The NPCs embedding pGNPs induced the recovery of injured neurons in SCI rats (Figure 3G). Considering the neuron‐inducing effects of pGNPs (Figure 2I) and the decreased GFAP‐expression levels around the lesions (Figure 3H) in the NPC‐pGNP group, NPCs embedding pGNPs may induce recovery of the injured neurons. The NPCs embedding pGNPs also induced the recovery of motor function after SCI in rats (Figure 3P). Therefore, we suggest that the transplantation of pGNP‐embedded NPCs derived from the embryonic spinal cord can be effective therapy for the repair of SCI.

## CONFLICT OF INTEREST

The authors declare no competing financial interest.

## Supporting information

Supporting Information
