# Supplementary material for: Neuron‐inducing therapy using embryonic neural progenitor cells embedding positively charged gold nanoparticles in rats with complete spinal cord injury
Source: Clin Transl Med. 2022 Jul 15;12(7):e981. doi: 10.1002/ctm2.981 (PMC9286526; doi:10.1002/ctm2.981)
Supplement: Supplementary file 1 — Supporting Information [file CTM2-12-e981-s001.docx]

**Supporting Information for**

**Neuron-inducing therapy using embryonic neural progenitor cells embedding positively charged gold nanoparticles in rats with complete spinal cord injury**

**Running Head: pGNPs induce neuronal differentiation from NPCs**

Gong Ho Han^1, 2†^, Wan-Kyu Ko^1, 2†^, Seong Jun Kim^1, 2^, Daye Lee^1, 2^, Dabin Jeong^1,3^, Inbo Han^1^, Seung Hun Sheen^1^, Seil Sohn^1*^

^1^Department of Neurosurgery, CHA Bundang Medical Center, CHA University, 59, Yatap-ro, Bundang-gu, Seongnam-si, Gyeonggi-do, 13496, Republic of Korea

^2^Department of Life Science, CHA University, 335, Pangyo-ro, Bundang-gu, Seongnam-si, Gyeonggi-do, 13488, Republic of Korea

^3^Department of Biology, Lawrence University, 711 E. Boldt Way, Appleton, WI 54911, USA

***Corresponding author:**

Seil Sohn, MD, PhD

Department of Neurosurgery,

CHA University College of Medicine

59, Yatap-ro, Bundang-gu, Seongnam-si, Gyeonggi-do, 13496, Korea

Phone: 82-31-881-7966

Fax: 82-2-780-5269

E-mail: sisohn@cha.ac.kr

^†^ Gong Ho Han and Wan-Kyu Ko contributed equally as the first authors

**MATERIALS AND METHODS**

**Materials**

Chloroauric acid (HAuCl_4_), trisodium citrate, and branched polyethyleneimine (BPEI) were purchased from Sigma Aldrich (St. Louis, MO). Neurobasal plus medium (GIBCO, Grand Island, NY USA) containing the B-27 supplement (GIBCO) and 1% penicillin-streptomycin (PS, GIBCO) was used as a cell culture medium. Dulbecco’s phosphate-buffered saline (DPBS) was purchased from GIBCO. Poly-D-lysine was obtained from GIBCO. The gC (molecular weight (MW): 50,000 Da) and sodium periodate (NaIO_4_) used here were purchased from Sigma. Sodium hyaluronate (HA, MW: 1,000 kDa) was provided by Humedix (Anyang, Korea).

**Synthesis of the negatively charged gold nanoparticles (nGNP)/positively charged GNP (pGNP) and characteristics evaluation**

The nGNP was prepared as previously described.^1,2^ HAuCl_4_ powder (136 mg) was dissolved in 800 mL of deionized water. The citrate powder (300 mg) was mixed with 15 mL of deionized water to make 2% citrate solution. The HAuCl_4_ solution was refluxed at 110 °C and the 15 mL of citrate solution was quickly added to the HAuCl_4_ solution. After 20 min, the color of the solution became dark red, containing 32 nm of nGNP.

The pGNP was prepared as described in the literature.^3^ Briefly, 9.72 mg of HAuCl_4_ powder was dissolved in 200 μL of deionized water. BPEI (350 mg) was diluted in 5 mL of deionized water to obtain 70 mg/mL of a polyethylenimine (PEI) solution, after which 87.45 μL of the PEI solution was transferred to 10 mL of deionized water. The HAuCl_4_ solution was slowly dropped into a PEI solution and gently stirred for seven days. The resulting dark red solution was stored at 4 ℃ before use. The Beer-Lambert law was used to calculate the concentrations of the synthesized nGNP/pGNP.^4,5^ The Ultraviolet-Visible (UV-Vis) spectroscopy (UV-1650PC, Shimadzu, Japan) and dynamic light scattering (DLS, Malvern 4700, Malvern, Worcestershire, UK) were used to measure the sizes of the nGNP/pGNP. The zeta potential (Zetasizer 2000, Malvern, Worcestershire, UK) values of the nGNP (0.1 nM)/pGNP (0.1 nM) were measured to determine the surface charges. Results are expressed as the mean ± standard error of the mean (SEM) (n = 5/group). The n is technical replicates.

**Neural progenitor cells (NPCs) isolation and storage**

The green fluorescent protein (GFP)-expressing spinal cord of embryonic day 14 (E14) was dissociated as described in the literature.^6^ Briefly, spinal cords were digested in 0.2% trypsin (GIBCO) for 10 minutes at 37 ℃, dissociated in medium, filtered using a 40 μm cell filter strainer, centrifuged, and then resuspended with a NPC stock solution (5 × 10^6^/vial; Cat. 05838; STEMCELL). The NPC vials were stored in liquid nitrogen.

**Coating method using poly-D-lysine for confocal dishes**

Poly-D-lysine in sterile Dulbecco’s phosphate-buffered saline (DPBS) was diluted to prepare a 50 µg/mL solution. We coated the surface of the confocal dishes with the solution of poly-D-lysine and incubated at room temperature for 1 h. After 1 h, the confocal dishes were washed three times with DPBS.

**Cytotoxicity test**

A cytotoxicity test was conducted using a cell viability assay kit (EZ-Cytox, Daeil Labservice, Korea), as previously described.^7^ NPCs were seeded onto a 24-well culture plate coated with poly-D-lysine at a density of 1 × 10^5^ with medium. The NPCs were cultured with medium containing 0.1 nM of nGNP or pGNP (n = 6 per group). After two days, the medium was removed, and the NPCs were incubated with a fresh medium containing the cell viability kit solution (500 μL of 0.1 mL/mL) for 1 h. Absorbances were measured at 450 nm using a microplate reader (Bio-Rad, Hercules, CA, USA).

**Dark field assay**

NPCs were seeded into 2 × 10^5^ cells on a poly-D-lysine coated confocal dishes. The NPCs were cultured with medium containing 0.1 nM nGNP or pGNP for 48 h. The nGNP or pGNP-treated NPCs were washed with DPBS and immobilized with 4% paraformaldehyde at room temperature for 10 min. The images were detected using an Olympus Optical microscope (BX53F2; Olympus, Japan).

**Immunocytochemistry (ICC) staining**

NPCs were seeded onto poly-D-lysine coated confocal dishes at a density of 2 × 10^5^ cells (n = 5 per group). One day after seeding, the NPCs were cultured for 48 h in medium containing 0.1 nM of nGNP or pGNP. After 48 h of incubation, cells were fixed with 4% paraformaldehyde. Fixed cells were incubated with neuron-specific class III beta-tubulin (Tuj-1) and the glial fibrillary acidic protein (GFAP) antibodies at 4 ℃ overnight. Primary antibodies for ICC staining were rabbit anti-Tuj-1 (1:200; Cat. ab221935; Abcam) and mouse anti-GFAP (1:200; Cat. MA5-12023; Abcam). Afterwards, Alexa 488 (1:500; Cat. A11034; Invitrogen) and Alexa 647 (1:200; Cat. A21236; Invitrogen) were incubated for 2 h at room temperature. Then, the nuclei of cells were stained with 4′,6′-diamidino-2-phenylindole dihydrochloride (DAPI). The stained NPCs were detected using a Zeiss LSM 880 confocal microscope.

**Fluorescence-activated cell sorting (FACS)**

The GFP-expressing embryonic-spinal-cord-derived NPCs were detached with ice-cold phosphate-buffered saline (PBS) containing 0.02% ethylenediaminetetraacetic (EDTA). 1×10^6^ GFP-expressing NPCs were incubated with primary antibodies, including anti-Pax6 (Cat. A7334; ABclonal Technology) and anti-Sox2 (Cat. GTX627404; GeneTex) for 1 hour. After one washing step, NPCs per 1 mL of stain buffer were stained with 5 uL Alexa 647 (Cat. A31571; Invitrogen) and Alexa 647 (Cat. A31573; Invitrogen). The marker expression was analysed on CytoFLEX V5-B5-R3 Flow Cytometer (Beckman Coulter), and data were analysed using the Kaluza software (Beckman Coulter).

**Neurite growth of NPCs in block regions**

NPCs were seeded at a density of 2 x 10^4^ cells onto a poly-D-lysine-coated SPL Scar Block (n = 5 per group, Cat. 201936, SPL Life Sciences, Pocheon, Republic of Korea). One day after seeding, the block was removed. The NPCs were cultured with medium containing 0.1 nM of nGNP (nGNP group) or pGNP (pGNP group). After seven days, the neurite growth was observed by means of Tuj-1 staining. Within the block region, rectangular region of interest (ROI, 17 × 16 mm^2^, n = 5 per group) were randomly designated for quantification by a blinded investigator with extensive experience in SCI experiments. The ‘n’ is the number of randomly designated ROIs. The Tuj-1 intensities in the ROI were quantified using ImageJ software (National Institutes of Health (NIH), Bethesda, MD, USA). The n is the number of randomly designated ROIs. Multiple comparisons were performed with ANOVA statistical test.

**CHA gel preparation**

The CHA gel was prepared as previously described.^1,8^ The gC was obtained from Sigma. The oHA was synthesized through an oxidation process using sodium periodate (NaIO_4_). The HA (3.8 g) was dissolved in deionized water (360 mL). The sodium periodate (1.068 g) was dissolved in a separate bottle of deionized water (40 mL). The dissolved sodium periodate was slowly added to the HA in a dark room. The mixture was stirred for 24 h and 1 mL of ethylene glycol (Sigma) was added to the mixture to neutralize the unreacted sodium periodate. The resultant solution was dialyzed for seven days using dialysis membranes (Spectrum Spectra, molecular weight cut off (MWCO): 12–14K). After dialysis, the oHA solution was lyophilized. The 2% gC and 3% oHA were separately dissolved in DPBS and centrifuged for 30 seconds at 13,000 rpm to remove bubbles. Then, the samples were stored at –20 °C before the use. In vivo, 2% gC and 3% oHA were used for the transplantation of NPCs. The volume ratio of the 2% gC and 3% oHA to form the CHA gel was 9:1.

**Transplantation of pGNP-embedded NPCs and injection of biotinylated dextran amine (BDA) into spinal cord injury (SCI) Sprague-Dawley (SD) rats**

Adult female SD rats (n = 18, 210-250 g, KOATECH, Seoul, Korea) and female Fisher 344 (F344) rats (n = 56, Rat Resource and Research Center, University of Missouri, Columbia, MO) were used in this study. All animal experimental procedures were conducted according to the regulations of the Institutional Animal Care and Use Committee of CHA University (IACUC200217) and according to the Guide for the Care and Use for Laboratory Animals (NIH).

SCI was induced at the exposed spinal cord following laminectomy of thoracic 10 (T10) using a complete compression method.^9^ The 50 mg/kg of Zoletil (Virbac Laboratories, Carros, France) and 10 mg/kg of Rompun (Bayer Animal Health Co, Suwon-si, Korea) were intraperitoneally injected to anesthetize the rats. A midline incision was made on the back, and laminectomy at the T10 level was performed to expose the dura. Spinal cord injuries were established by completely compressing method using Dumont #2 forceps (#11223-20; Fine Science Tools). Tract-tracing of axons was performed via a BDA (MW: 10,000, Cat. D1956; Invitrogen) injection. BDA was injected into two sites of an intact region (one on each side of the cord, 0.5 μL/site) at a region 1.5 mm below the surface at a rate of 0.1 μL/minute using a 33-gauge Hamilton syringe (Cat. 7635-01; Hamilton). Transplantation of pGNP-embedded NPCs was performed seven days after SCI.

After seven days, NPCs embedded with CHA gel containing 0.1 nM of pGNP were transplanted at three sites of the spinal cord, specifically the lesion epicenter (1st spot), 1 mm in the rostal direction (intact region, 2nd spot), and 1 mm in the caudal direction (intact region, 3rd spot). Using a 31-gauge microliter syringe (Cat. 7639-01; Hamilton), an amount of 5 μL of NPC (1×10^6^ cells per site) graft containing pGNP (0.1 nM) was transplanted into the three pre-determined sites.

All rats were housed in a pathogen-free facility with controlled temperature and humidity levels. Rats were allowed free access to food. The bladders of the SCI rats were manually emptied several times daily until normal function returned.

**Quantitative real-time polymerase chain reaction (qRT-PCR)**

Four weeks after transplantation of the NPCs, all spinal-cord-injured rats were sacrificed for qRT-PCR.^9^ RNA from 10 mm spinal cord segments including the lesion epicenter was extracted using Trizol reagent (Invitrogen) according to the manufacturer’s instructions. Complementary DNA (cDNA) was synthesized from 1 μg of the total RNA using Maxim RT Premix kit (iNtRON Biotechnology, Inc., Korea). The qRT-PCR step was performed with an ABI Step-One real-time PCR system (Applied Biosystems, Warrington, UK). The reaction mixture consisted of SYBR Green 2X PCR Master Mix (Applied Biosystems), a cDNA template, and forward/reverse primers. The relative expression levels of Tuj-1, GFAP, and Neurofilament-heavy (NF-H) were normalized to that of glyceraldehyde 3-phosphate dehydrogenase (GAPDH) using $2^{-\Delta\Delta C_{T}}$method. The primers were obtained from Bioneer (Daejeon, Korea). The primer sequences used in this study are shown in Table S1. The comparisons among the Injury, NPC, and NPC-pGNP groups at 35-day post injury were conducted with a one-way analysis of variance (ANOVA) and Tukey's multiple-comparison test was used as a post-hoc analysis method.

**Immunofluorescence (IF) staining and quantification in vivo**

Four weeks after transplantation of the NPC, all spinal-cord-injured rats were perfused for IF staining as previously described.^1^ Spinal cord segments of 10 mm were fixed in 4% paraformaldehyde. They were then dehydrated and embedded with paraffin as previously described.^7,10^ Tuj-1, GFP, GFAP, neuronal nuclei (NeuN), and BDA antibodies were used for IF staining.

Primary antibodies were rabbit anti-Tuj-1 (1:200; Cat.ab221935; Abcam), mouse anti-GFAP (1:200; Cat. MA5-12023; Invitrogen), mouse anti-NeuN (1:200; Cat. MAB377; Merk), and rabbit anti-GFP (1:200; Cat. A11122; Invitrogen), Goat anti-NeuroD1 (1:200; Cat. AF246, R&D system), mouse anti-Nestin (1:200; Cat. GTX630201, GeneTex), and rabbit anti-Pax6 (1:200; Cat. A7334, ABclonal Technology). Fluorescence secondary antibodies were Alexa 488 (1:500; Cat. NBP2-22111AF488; NOVOUS), Alexa 488 (1:500; Cat. A11034; Invitrogen), Alexa 647 (1:500; Cat. A21236; Invitrogen), and Alexa 594 (1:500; Cat. S32357; Invitrogen).

According to standard procedures for IF staining, deparaffinized tissue sections were incubated in a blocking solution to prevent any non-specific binding reaction for 1 h. The primary antibodies used here were Tuj-1, GFAP, and NeuN. They were incubated at 4 °C overnight. The sections were then stained with fluorescent secondary Alexa 488, Alexa 647, or Alexa 594 (1:500; Invitrogen) for 2 h at room temperature. Afterwards, the nuclei were stained with DAPI. Sections were washed in PBS and then mounted with a specific medium (DakoCytomation, Glostrup, Denmark). Confocal images were acquired using a Zeiss LSM 880 confocal microscope.

ROIs were designated for quantification. The ROIs are shown as tiled scan images. The ROI for NeuN/GFP/DAPI were 500 × 460 μm^2^ (n = 6 per group). The ROIs for GFAP/Tuj-1/DAPI and BDA/GFAP/DAPI were 470 × 430 μm^2^ (n = 6 per group). The n is the number of randomly designated ROIs.

The blinded investigator randomly selected ROIs for the IF measurement. Within the ROI, NeuN-stained intensities were divided with the GFP intensities for quantification using ImageJ software (NIH). Within the ROI, Tuj-1, GFAP, and BDA-stained intensities were divided with the DAPI intensities for quantification using ImageJ software (NIH), as previously described.^10,11^ Multiple comparisons were performed with ANOVA statistical test.

**Behavioral tests**

Basso, Beattie, Bresnahan (BBB) is a 22-point scale (with scores of 0-21) that systematically and logically follows the recovery of hindlimb function from a score of 0, indicative of no observed hindlimb movements, to a score of 21, representative of a normal ambulating rodent. To measure the scores of the SCI rats, a circular metal enclosure (106.7 cm diameter, 61 cm wall height) was used. The SCI rats were exposed to the testing environment at day 0, 1, 3, 5, 7, 14, 21, 28, and 35. BBB scores were obtained from two investigators with extensive experience according to the BBB criteria.^1^ The investigators were blinded to this study. Four SD rats per group after SCI were randomly observed at 0, 1, 3, 7, 14, 21, 28, and 35 days (n = 6 per group).

**Statistical analyses**

Multiple comparisons between groups were performed by a one-way analysis of variance (ANOVA), and Tukey’s multiple comparisons test was used as post-hoc analysis method. Behavioral scores were analyzed by Student’s t-test. Differences with **p < 0.05*, ***p < 0.01*, and ****p < 0.001* were considered as statistically significant.

**Supplementary Figures**


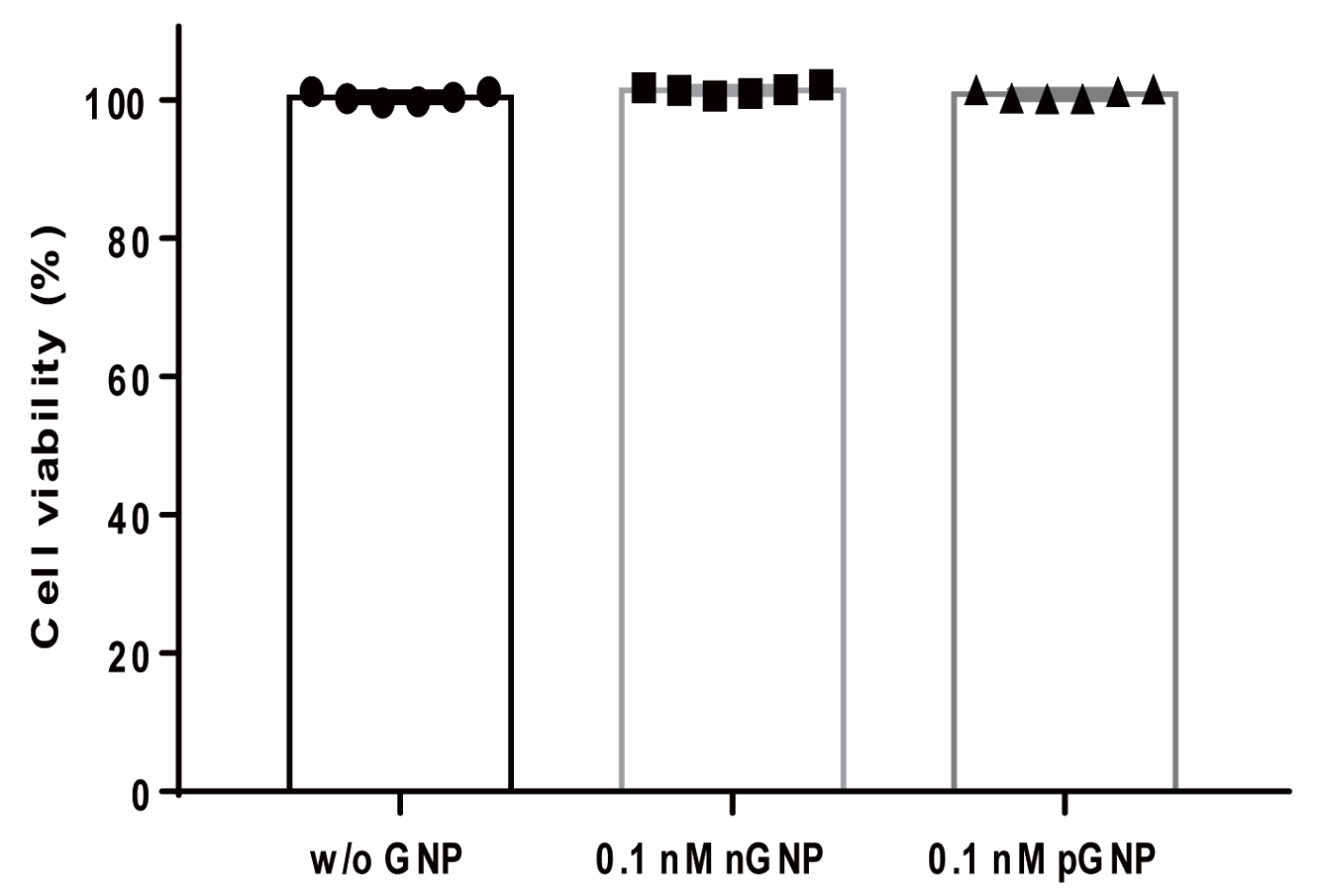


**FIGURE S1** Cytotoxicity test of without GNP, nGNP, and pGNP into NPCs for 48 h. The NPCs were treated with 0.1 nM of nGNP or pGNP for 48 h. NPCs viability of the without GNP, nGNP, and pGNP-treated groups was measured using a CCK-8 assay (n=6).


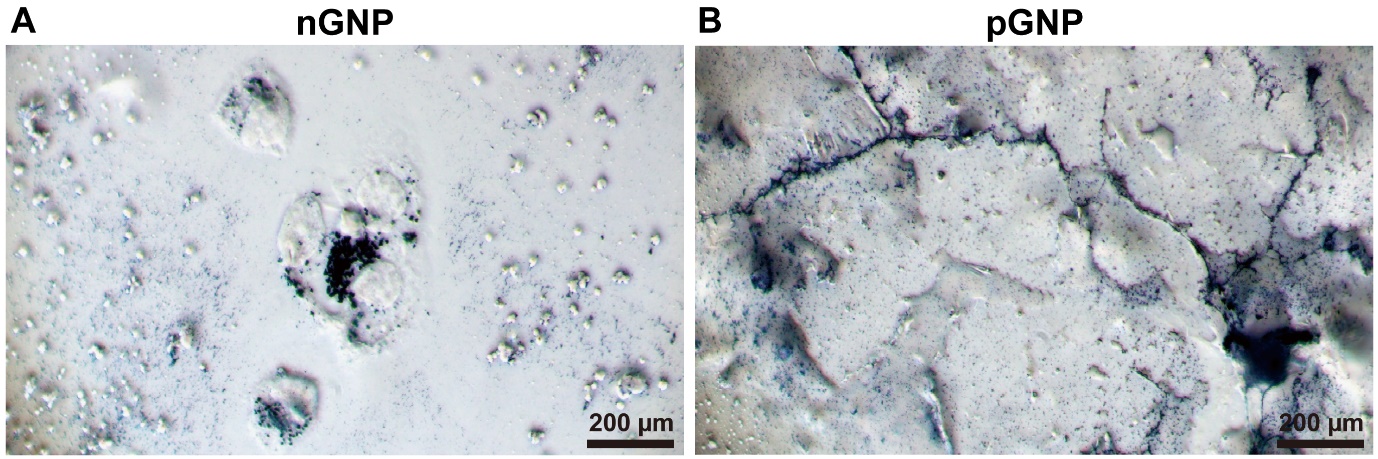


**FIGURE S2** The cellular uptake of nGNP and pGNP into NPCs for 48 h. Representative images of NPCs were treated 0.1 nM of (A) nGNP or (B) pGNP for 48 h. Scale bar is 200 μm.

**
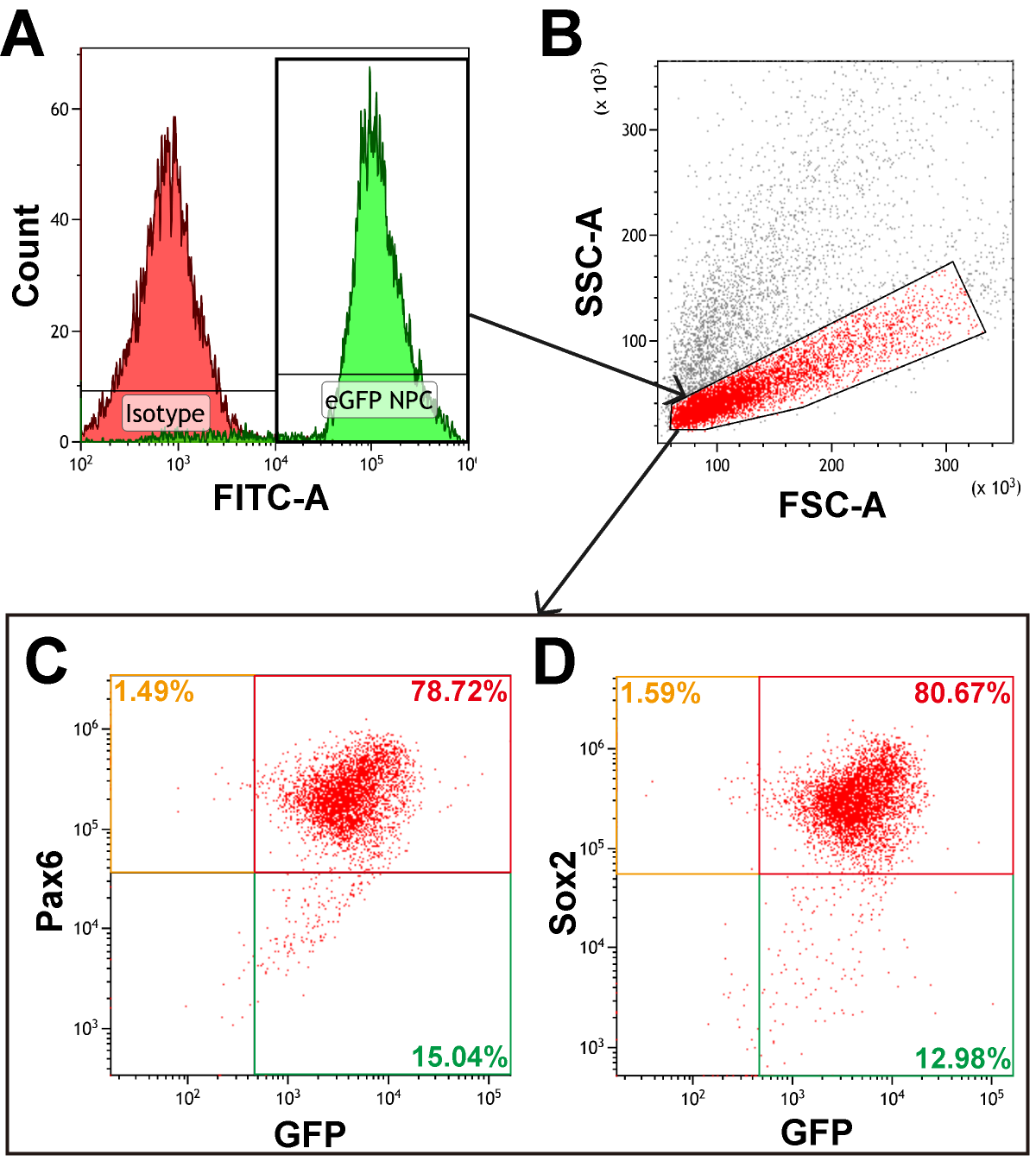
**

**FIGURE S3** Fluorescence-activated cell sorting (FACS) of embryonic-spinal-cord-derived neural progenitor cells (NPCs). (A-B) Representative forward scatter (FSC) and side scatter (SSC) plots showing the gate based on GFP-expressing NPCs. This strategy produced the following NPCs populations: Within (C) GFP-/Pax6-, GFP+/Pax6-, GFP-/Pax6+, and GFP+/Pax6+. Within (D) GFP-/Sox2-, GFP+/Sox2-, GFP-/Sox2+, and GFP+/Sox2+.

**
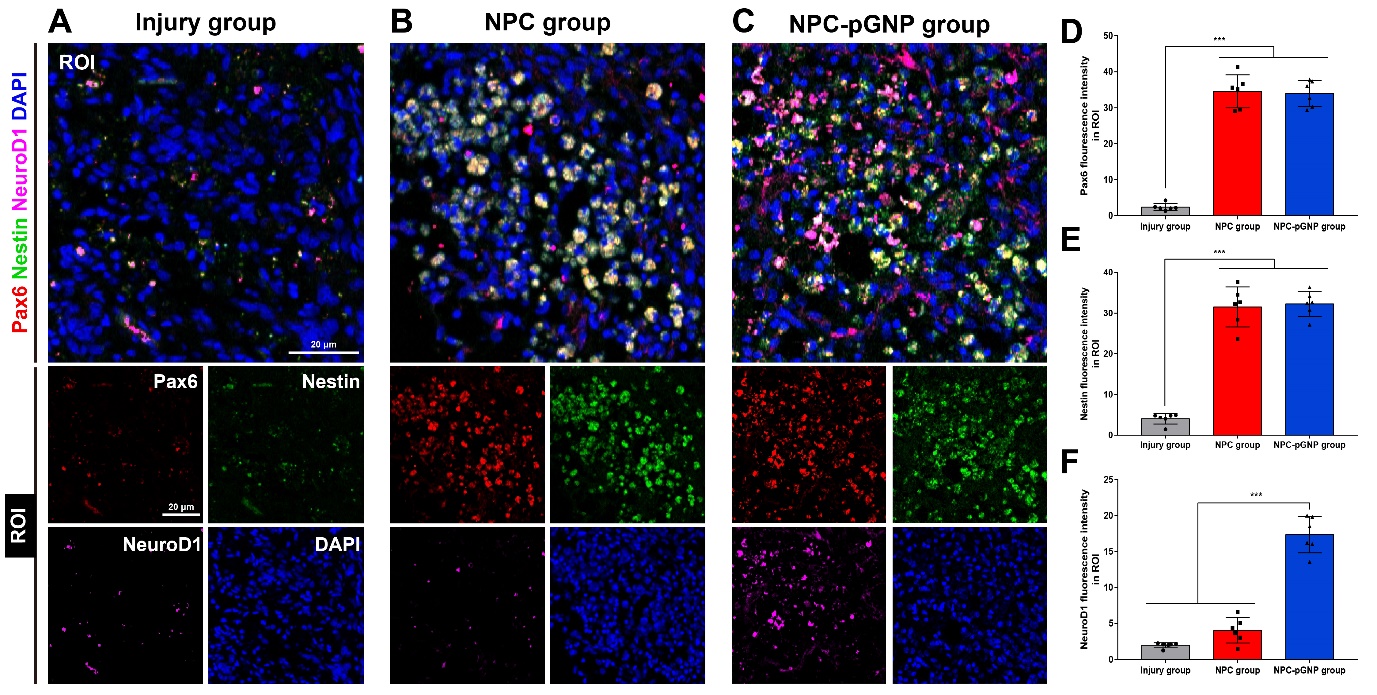
**

**Figure S4** Immunofluorescence (IF) staining. Representative ROI images in the (A) Injury group, (B) NPC group, and (C) NPC-pGNP group. (D) Quantitative analysis of Pax6 intensity in the ROI. (E) Quantitative analysis of Nestin intensity in the ROI. (F) Quantitative analysis of NeuroD1 intensity in the ROI. Results are expressed as the mean ± SEM (n = 6/group, *^***^p < .001*).

**
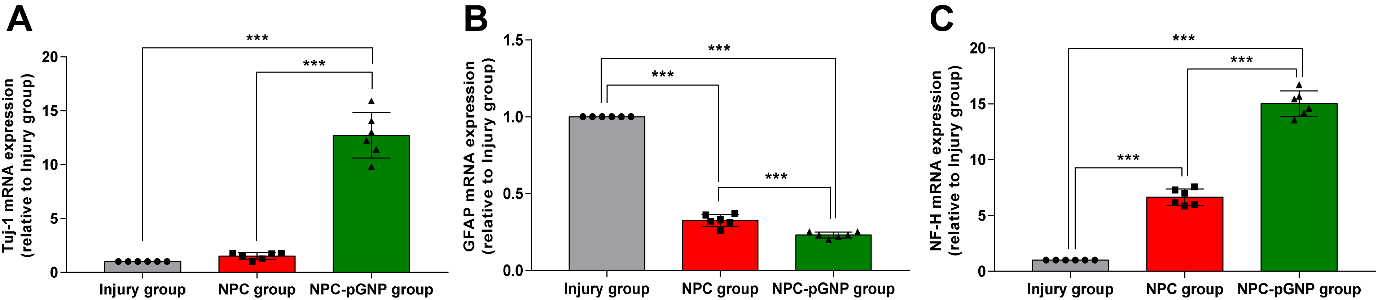
**
**FIGURE S5** The (A) Tuj-1, (B) GFAP, and (C) NF-H mRNA expression values in the Injury, NPC, NPC-pGNP groups. The results are expressed as the mean ± SEM (n = 6/group, *^***^p < .001*).

**Table S1** Nucleotide sequences of primers used in qRT-PCR

| **Gene** | **Forward (5' - 3')** | **Reverse (5' - 3')** |
| --- | --- | --- |
| **Tuj-1** | **GCGATGCTACCGCAAAGAAC** | **ATGGCGTCTCCCACACTACC** |
| **GFAP** | **CTGGTGTGGAGTGCCTTCGT** | **CACCAACCAGCTTCCGAGAG** |
| **NF-H** | **AGGACCTGCTCAACGTCAAGAT** | **TCCAAAGCCAATCCGACACT** |
| **GAPDH** | **GCCATCAACGACCCCTTCAT** | **TTCACACCCATCACAAACA** |

**References**

1. Ko WK, Lee SJ, Kim SJ, et al. Direct Injection of Hydrogels Embedding Gold Nanoparticles for Local Therapy after Spinal Cord Injury. *Biomacromolecules.* 2021;22:2887-2901.

2. Ko WK, Heo DN, Moon HJ, et al. The effect of gold nanoparticle size on osteogenic differentiation of adipose-derived stem cells. *J Colloid Interface Sci.* 2015;438:68-76.

3. Wen S, Zheng F, Shen M, Shi X. Synthesis of polyethyleneimine-stabilized gold nanoparticles for colorimetric sensing of heparin. *Colloids Surf Physicochem Eng Aspects.* 2013;419:80-86.

4. Lee D, Ko W-K, Kim SJ, et al. Inhibitory Effects of Gold and Silver Nanoparticles on the Differentiation into Osteoclasts In Vitro. *Pharmaceutics.* 2021;13:462.

5. Rahman S. Size and concentration analysis of gold nanoparticles with ultraviolet-visible spectroscopy. *Undergrad J Math Model One+ Two.* 2016;7:13.

6. Lu P, Graham L, Wang Y, Wu D, Tuszynski M. Promotion of survival and differentiation of neural stem cells with fibrin and growth factor cocktails after severe spinal cord injury. *J Vis Exp.* 2014:e50641.

7. Lee D, Nah H, Ko W-K, et al. Thiolate poly (lactic-co-glycolic acid) nanofibers loaded with dexamethasone and ropivacaine show enhanced sustained release in the treatment of neuropathic pain through a local therapy technique. *Chemical Engineering Journal.* 2022;431:133356.

8. Han GH, Kim SJ, Ko WK, et al. Injectable Hydrogel Containing Tauroursodeoxycholic Acid for Anti-neuroinflammatory Therapy After Spinal Cord Injury in Rats. *Mol Neurobiol.* 2020.

9. Anderson MA, O'Shea TM, Burda JE, et al. Required growth facilitators propel axon regeneration across complete spinal cord injury. *Nature.* 2018;561:396-400.

10. Han GH, Kim SJ, Ko WK, et al. Transplantation of tauroursodeoxycholic acid-inducing M2-phenotype macrophages promotes an anti-neuroinflammatory effect and functional recovery after spinal cord injury in rats. *Cell Prolif.* 2021;54:e13050.

11. Han GH, Kim SJ, Ko WK, et al. Injectable Hydrogel Containing Tauroursodeoxycholic Acid for Anti-neuroinflammatory Therapy After Spinal Cord Injury in Rats. *Mol Neurobiol.* 2020;57:4007-4017.
